# Optimum deposition conditions of ultrasmooth silver nanolayers

**DOI:** 10.1186/1556-276X-9-153

**Published:** 2014-03-31

**Authors:** Tomasz Stefaniuk, Piotr Wróbel, Ewa Górecka, Tomasz Szoplik

**Affiliations:** 1 Faculty of Physics, University of Warsaw, Pasteura 7, Warsaw 02-093, Poland; 2 Department of Chemistry, University of Warsaw, Żwirki i Wigury 101, Warsaw 02-089, Poland

**Keywords:** Thin films, Plasmonics, Roughness, Physical vapor deposition, Nanooptics

## Abstract

Reduction of surface plasmon-polariton losses due to their scattering on metal surface roughness still remains a challenge in the fabrication of plasmonic devices for nanooptics. To achieve smooth silver films, we study the dependence of surface roughness on the evaporation temperature in a physical vapor deposition process. At the deposition temperature range 90 to 500 K, the mismatch of thermal expansion coefficients of Ag, Ge wetting layer, and sapphire substrate does not deteriorate the metal surface. To avoid ice crystal formation on substrates, the working temperature of the whole physical vapor deposition process should exceed that of the sublimation at the evaporation pressure range. At optimum room temperature, the root-mean-square (RMS) surface roughness was successfully reduced to 0.2 nm for a 10-nm Ag layer on sapphire substrate with a 1-nm germanium wetting interlayer. Silver layers of 10- and 30-nm thickness were examined using an atomic force microscope (AFM), X-ray reflectometry (XRR), and two-dimensional X-ray diffraction (XRD2).

**PACS:**

63.22.Np Layered systems; 68. Surfaces and interfaces; thin films and nanosystems (structure and nonelectronic properties); 81.07.-b Nanoscale materials and structures: fabrication and characterization

## Background

Surface plasmon-polariton (SPP) waves excited on a metal-dielectric interface allow the control and manipulation of light at nanoscale dimensions [1]. The propagation range of SPPs on a metal-dielectric interface is limited due to ohmic losses and scattering on random and intended interface irregularities [2-4]. Ohmic losses of free electrons depend on the SPP frequency range and the temperature of the structure and thus cannot be ultimately reduced. Therefore, further development of plasmonic devices is possible via reduction of scattering losses of SPPs. In order to limit scattering on a patterned metal surface, an idea to control the flow of light on a metal-dielectric interface through structuring the dielectric layer or modification of the permittivity tensor of the dielectric side of the structure was proposed [5,6]. However, random surface roughness and metal islands induce scattering on both structured and flat surfaces and thus deteriorate functioning of plasmonic devices [7-9].

It was shown in experiments that surface plasmon losses in various plasmonic structures are virtually insensitive to temperature change. A PMMA/Ta_2_O_5_/Au multilayer on glass substrate has almost the same transmission spectrum at wavelength range 550 to 800 nm measured in temperatures from 80 to 350 K [10]. The decrease of electrical resistivity of silver with the reduction of temperature does not influence the surface plasmon loss. The imaginary part of electric permittivity of silver, which is inversely proportional to the ohmic conductivity, changes with temperature but depends mostly on the silver film thickness. Thus, it is not the ohmic losses due to electron scattering in silver but the temperature-independent morphology of the silver surface that decides on losses due to scattering into free space [2]. The above conclusion is in agreement with recently observed maxima in the visible range of the transmittance spectra of Ag/MgF_2_/Ag [11], Ag/ITO/Ag [12], and ZnO/Ag/ZnO [13] multilayers, which clearly depend on Ag surface morphology.

Heteroepitaxial deposition of ultrasmooth noble metal layers on crystalline or glass substrates is described with one of two ideal growth manners. In the Frank-van der Merwe deposition mode, the process begins with atom-thick islands, which dilate, connect, and eventually form continuous layers. In the Stranski-Krastanov (SK) growth, after the first few layers are formed, the nucleation of island begins because of strains and diffusivity of adatoms. In electron beam deposition processes, an atom evaporating from a hot crucible (about 1,200 K) arrives onto a substrate kept at room temperature (RT) and slowly loses its kinetic energy. Diffusivity of metal adatoms on the surface diminishes with decreasing substrate temperature. Thus, cooling the substrates to cryogenic temperatures should in principle lead to ultrasmooth layers.

The role of surface diffusivity of Ag adatoms in the formation of islands and then grains was demonstrated by Jing et al. in STM measurements, where with increasing layer thickness the silver clusters were more and more pronounced [14]. The same authors observed that deposition of 12 monolayers of silver at 190 K results in an increase of island densities by 4 orders of magnitude in comparison to that obtained at RT. At the same time, silver atom clusters were at least 1 order of magnitude smaller. The diffusivity of Ag adatoms is reduced with an amorphous 1-nm Ge interlayer [15-17], 5-nm layer of chromium [18], or 1-nm film of Ti [19]. A 2-nm nickel layer is nearly as efficient in metal layer smoothing as germanium, which moreover enhances the surface plasmon resonance sensitivity of the Ag layer [20]. Recently, a 1-nm-thick copper seed layer was also reported to be effective in smoothing silver nanolayers [21]. When a continuous 6-nm Ag layer on 1 nm of Ge is sequentially deposited on fused silica substrate without breaking the chamber vacuum, a silver surface roughness of root-mean-square (RMS) = 0.6 nm is achievable [22]. In Ag/MgF_2_/Ag on quartz with a Ge seed growth layer, the roughness of the silver surface considerably modifies the reflectance spectra [11]. In our recent paper [19], we proved that the smoothness of Ag/Ge, Ag/Ni, and Ag/Ti films - that is, reduction of losses on scattering - is achieved at the cost of increased specific resistance - that is, increase of ohmic losses in the skin depth-thick layer of silver.

In this article, we discuss methods to achieve ultrasmooth silver nanolayers on sapphire substrate with germanium interlayer by optimizing the temperature for the range of evaporation pressures. Roughness results from island evaporation which is related to the surface diffusivity of Ag adatoms. Therefore, we investigate the influence of substrate temperature on the surface diffusivity of adatoms.

## Methods

### Electron-beam physical vapor deposition

We deposited polycrystalline silver films with an electron-beam evaporator (PVD75, Lesker, Hastings, UK). Epi-polished c-plane (0001)-oriented sapphire wafers with nominal roughness RMS = 0.2 nm were used as substrates. Before deposition, the substrates were bombarded with argon ions with 105 eV energy and 0.2 mA/cm^2^ beam density for 30 s. Before evaporation, both the substrate holder and the chamber walls were heated for 12 h at 420 and 330 K, respectively. A germanium adhesion layer (1 nm) and silver layers (10 and 30 nm) were sequentially evaporated at the same temperature and at a deposition rate equal to 0.05 nm/s without breaking the vacuum. To minimize absolute humidity (defined as the ratio of mass of water vapor to volume of vapor/air mixture) in the vacuum chamber, we reduced the pressure to the lowest achievable level 5 × 10^−8^ Torr. During the process of Ge and Ag evaporation lasting a few minutes, the pressure has increased by 1 order of magnitude. For the period of the deposition of films, the vacuum chamber was kept at RT and the temperature of a custom-made sample holder module was controlled in the range 90 to 500 K with 10^−1^ K accuracy. The upper part of the module had liquid nitrogen (LN2) temperature and worked as a cold trap, which reduced substrate contamination and improved the vacuum within the chamber. The temperature of the lower part was measured using two platinum sensors (PT-103, Lake Shore Cryotronics, Westerville, OH, USA), the first located inside the holder in a drilled channel and the second attached to the holder surface. For heating, a twin core wire with cold ends (Thermocoax, Suresnes, France) was used with regulated power supply (Cryogenic Temperature Controller 335, Lake Shore Cryotronics). During the deposition, the substrate temperature increased by, at most, 5° due to radiation from the crucible. After deposition, the cryostat and the samples reached RT in a natural heat exchange process lasting up to 12 h and then the chamber was filled with nitrogen. Before morphology characterization in ambient conditions, the samples were kept in an Ar (6 N) atmosphere.

### Scanned AFM images

Atomic force microscope (AFM) measurements under tapping mode in air were carried out utilizing an Ntegra NT-MDT microscope (Moscow, Russia) equipped with sharp etalon probes with 10-nm tip curvature radius and 5:1 aspect ratio. Such probes are characterized by highly reproducible parameters: typical dispersion of probe resonant frequency is ±10% and typical dispersion of force constant is ±20%. The resonant frequency of the probes is equal to 140 kHz, which corresponds to a force constant of 3.5 N/m. To calibrate AFM scanner movements along the *z*-axis, highly oriented pyrolytic graphite was used. Calibration in the lateral direction was performed using a three-dimensional array of rectangles with 3-μm period.

### X-ray reflectometry and diffractometry

The structure of thin films was analyzed by X-ray reflectometry; the measurements were performed using the Bruker Discover D8 X-ray diffractometer (Madison, WI, USA) with Cu Kα line source of wavelength 0.15405 nm and point detector. The monochromatic parallel beam was formed by a parabolic Goebel mirror. The data analysis was based on finding the proper electron density profile, whose Fourier transform would match the recorded X-ray reflectometry (XRR) pattern. To fit the data, a ‘box model’ was used. Data fitting was performed using Leptos 4.02 software package provided by Bruker. The thickness and density of Ag and Ge layers as well as Ge/Ag and Ag/air surface roughness were free parameters in the fitting procedure. The wide-angle X-ray diffraction (XRD) measurements were done with the Bruker GADDS system equipped with 2D Vantec 2000 detector.

## Results and discussion

### Effect of thermal expansion

Deposition of metal layers on cooled dielectric substrates poses a question about the relationship between the dimensional stability of structures and temperature change. A mismatch of thermal expansion coefficients of layers gives rise to intrinsic stress that may result in metal film cracking. The thermal expansion coefficient of silver *α*_Ag_ varies from 13.38 at 85 K to 18.8 [μm/m K] at RT [23]. At temperatures from 90 to 295 K, the expansion coefficient of sapphire *α*_sapphire_ in the (0001) plane increases from 3.3 to 6.5 [μm/m K] [24]. The temperature difference between the cooled substrates and RT (at which samples are usually removed from the vacuum chamber) can be as much as 200°.

To assess the influence of thermal expansion on deposited metal layers, the same Ag layer sample deposited at RT was characterized in two cases: once just after deposition and the second time after rapid cooling down to 90 K and heating back to RT in N2 flow in ambient conditions. In AFM images, we measured three surface morphology parameters of the sample: the ten-point height value given as the difference between five maximal peaks and five minimal hollows, average height value, and RMS roughness. In spite of LN2 cooling, both the granularity and roughness of the silver film remained nearly the same and the temperature change did not cause any cracks.

### Effect of cooling substrates

While thermal expansion of materials involved in the deposition process has a negligible influence on Ag film roughness, we decide to cool down the substrates and thus reduce the surface diffusivity of adatoms. The diffusivity of Ag adatoms was preliminarily reduced due to an intermediate 1-nm-thick wetting layer of germanium [15]. In the vacuum chamber during the deposition process, the specific humidity (defined as the ratio of mass of water vapor to unit mass of dry air) is kept constant in spite of the pressure decrease. However, when the substrate is rapidly cooled with LN2, this specific humidity considerably decreases because most of the water vapor condenses on cooled parts and freezes forming ice crystals of a size reaching single nanometers. In our custom-made substrate holder module, most of the residual humidity did not deposit on the substrates with controlled temperature but on the walls of the LN2 vessel, which was the coldest element in the vacuum chamber and worked as a cold trap. Nevertheless, silver was deposited on the ice crystal-covered substrate, which no longer has flatness RMS = 0.2 nm.

**Figure 1:**
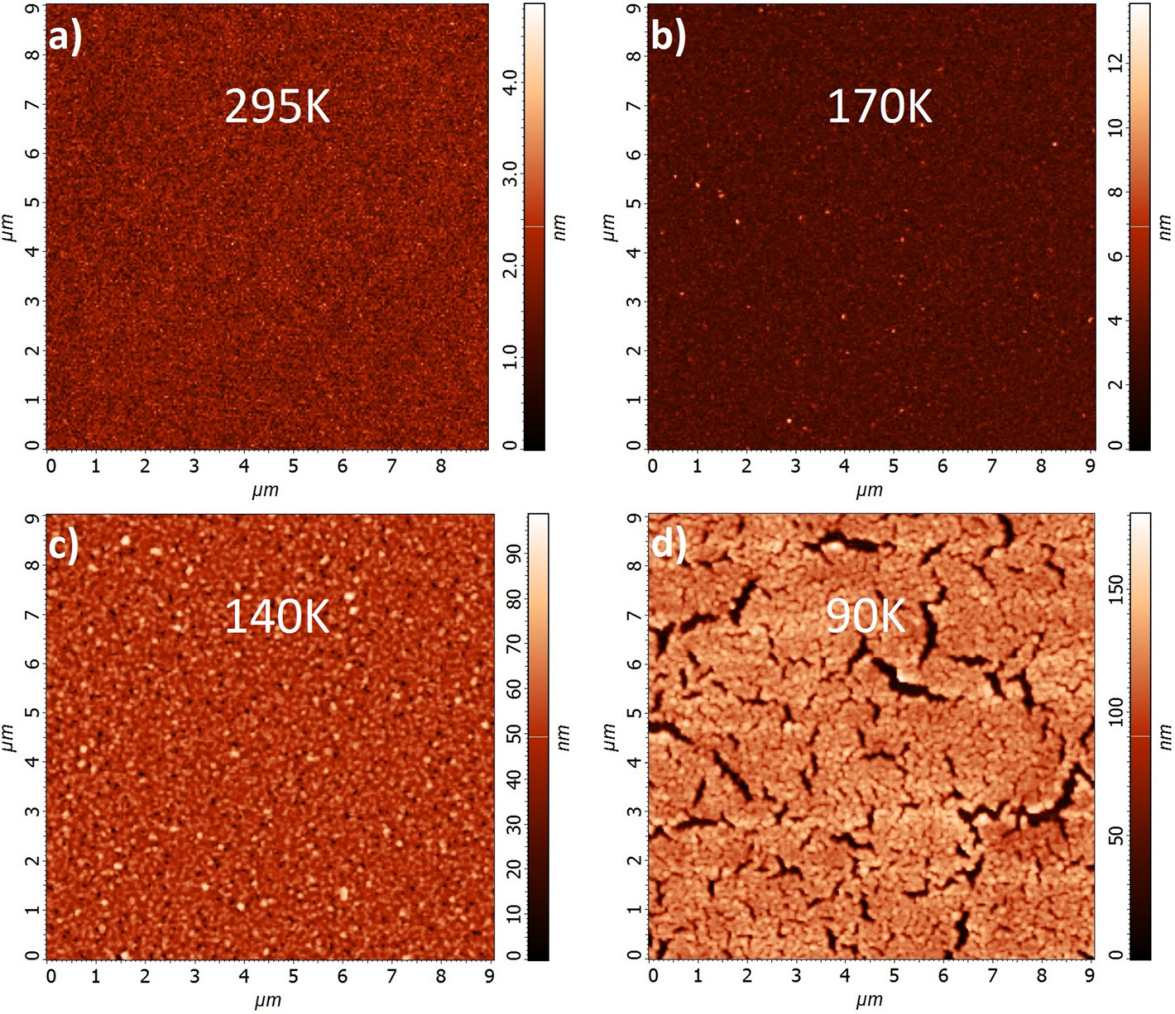
**AFM images of 30-nm-thick Ag films scanned at RT.** Samples deposited at **(a)** 295 K and **(b)** 170 K - the surface smoothness is influenced solely by thermal migration of atoms leading to continuous and almost uniform layers, **(c)** at 140 K - islands due to atom migration and deposition onto sapphire substrate covered with water ice nanocrystals are more pronounced, and **(d)** at 90 K - the surface smoothness is deteriorated by cracks that result from water ice crystal melting.

Now, we look for the optimum temperature of depositing 30-nm-thick Ag films at temperatures from the range 90 to 400 K. Figure 1 shows AFM images scanned on 9 × 9 μm areas of 30-nm-thick Ag films deposited at temperatures 295, 170, 140, and 90 K. Surface morphology parameters of the samples are given in Table 1. Films deposited at two high temperatures have comparable surface quality (Figure 1a, b); however, the ten-point height value is lowest in the sample deposited at ambient temperature (Figure 1a). The morphology parameters of the samples evaporated at the two low temperatures are poorer. Figure 1d shows that the silver film was deposited on water ice crystals. After melting of the crystals, some silver flakes are only loosely connected with the substrate. The rift valleys shown in Figure 1d are micrometers long and their deep end reaches the substrate.

**Table 1:** **AFM scan parameters of 9 × 9 μm**^**2 **^**area of 30-nm-thick Ag layer**

**Ag/Ge/Al**_**2**_**O**_**3**_	**295 K**	**170 K**	**140 K**	**90 K**
Ten-point height [nm]	2.04	6.79	50.5	112.3
Average height [nm]	1.73	3.65	40.96	90.88
RMS roughness [nm]	0.49	0.77	9.54	28.30

Thin Ag films were deposited on sapphire substrates with 1-nm Ge wetting layer at different temperatures.

**Figure 2:**
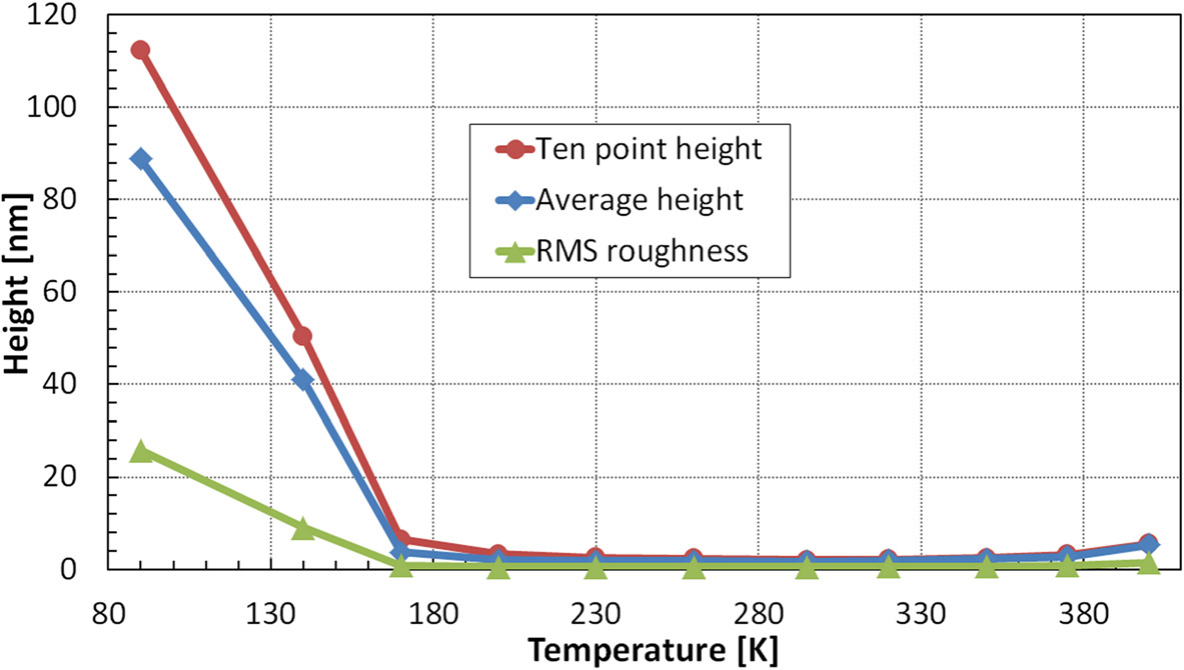
**Three surface morphology parameters measured using AFM on 3 × 3 μm**^**2 **^**area of 30-nm-thick Ag layers.** Thin Ag films were deposited on sapphire substrates with Ge wetting monolayer at temperatures in the range 90 to 400 K.

Figure 2 shows temperature-dependent plots of surface morphology parameters: ten-point height, average height, and RMS roughness values measured using AFM on 30-nm-thick Ag films. For deposition at temperatures above 170 K, the considered criteria values indicate that virtually any temperature from the range 230 to 350 K can be chosen. In 30-nm-thick films at temperatures below 230 K, the mobility of Ag adatoms is not high enough to assemble a uniform layer. A cohesive force between adatoms is not strongly manifested, and the position of the adatoms is determined by the point of arrival. On the contrary, at temperatures higher than 350 K, Ag adatoms have enough kinetic energy to migrate to the edge of the nearest island or even build up the next layer on top of it. The ten-point height criterion is crucial for assessment of scattering losses as both peaks and hollows act as strong scatterers. Deteriorated surfaces of Ag films deposited at temperatures below 170 K are connected with evaporating onto substrates covered with water ice nanocrystals.

### Effect of water ice crystallization

Cooling leads to the formation of water ice crystals on substrates at temperatures lower than sublimation phase transition at pressures below the water triple point in its phase diagram.

The recently formulated new sublimation-pressure empirical equation valid in the range from 50 K and 1.9 × 10^−34^ MPa to the triple point, where all three phases of water are in equilibrium at *T*_t_ = 273.16 K and *p*_t_ = (611.657 ± 0.010) Pa, is composed of three terms [25]

(1) $$\ln\pi =\theta^{-1}\sum_{i=1}^{3}a_{i}\theta^{b_{i}}$$

where *π* = *p*_subl_/*p*_t_ and *θ* = *T*/*T*_t_. The equation coefficients *a*_i_ and *b*_i_ are given in Table 2.

**Table 2:** Sublimation-pressure empirical equation coefficients

**Coefficient**	**Value**
*a*_1_	−0.212144006 × 10^2^
*a*_2_	0.273203819 × 10^2^
*a*_3_	−0.610598130 × 10^1^
*b*_1_	0.333333333 × 10^−2^
*b*_2_	0.120666667 × 10^1^
*b*_3_	0.170333333 × 10^1^

**Figure 3:**
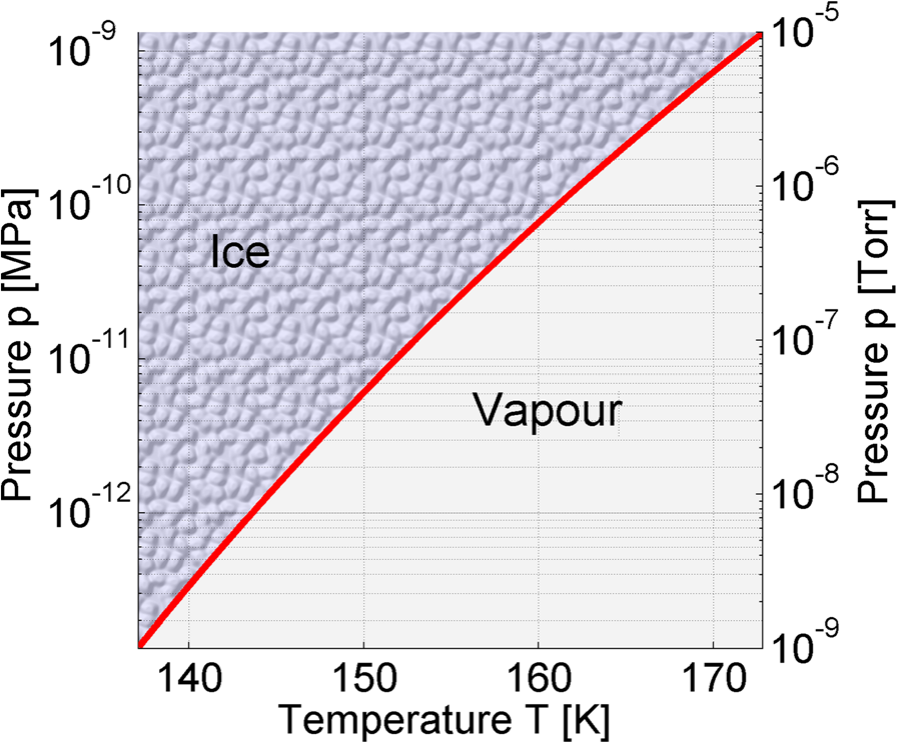
**Phase transitions of water.** The *p*-*T* diagram is calculated with the new sublimation-pressure empirical equation valid in the range from 50 K and 1.9 × 10^−40^ Pa to temperature and pressure values at the triple point [25]. The sublimation-pressure curve is delineated for pressures ranging from 10^−5^ Torr down to 10^−9^ Torr that are achievable in vacuum chambers of physical vapor deposition systems.

A *p*-*T* diagram with phase-boundary curves separating solid and gaseous forms of water within the temperature range 140 to 170 K is shown in Figure 3. It shows the sublimation-pressure curve for pressures ranging from 10^−5^ Torr down to 10^−9^ Torr, at which metals are deposited in e-beam evaporators. At 10^−8^ Torr, the sublimation temperature is 144.6 K, and at 10^−7^ Torr, it is 152.9 K.

To assure proper adhesion of the deposited material to a Ge-wetted substrate surface and to avoid water ice crystal growth, which leads to the increase of substrate roughness, the system should operate at the lowest possible pressures and all the time on the high temperature side of the *p*-*T* diagram shown in Figure 3.

### Optimum deposition temperature

**Figure 4:**
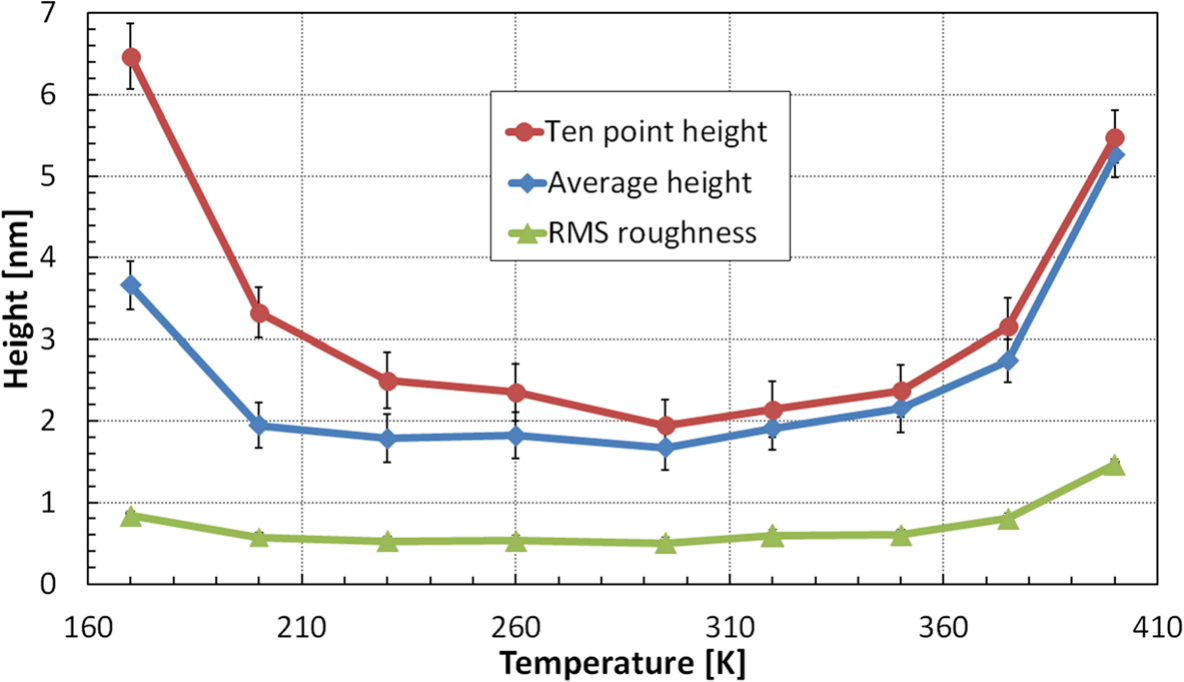
**Three surface morphology parameters measured using AFM on 3 × 3 μm**^**2 **^**area of 30-nm-thick Ag layers.** Thin Ag films were deposited on sapphire substrates with Ge wetting monolayer at temperatures in the range 170 to 400 K.

Figure 4 shows temperature-dependent plots of surface morphology parameters: ten-point height, average height, and RMS roughness values measured using AFM on 30-nm-thick Ag films for deposition at temperatures above that of sublimation. Notice the vertical scale different from that in Figure 2. Within the range 230 to 350 K, RMS roughness has nearly the same value. Two other criteria have minimum values at RT.

The morphology of crystalline 30-nm-thick Ag layers was analyzed using two-dimensional X-ray diffraction (XRD2). The XRD2 pattern from one of the 30-nm-thick Ag samples deposited at 295 K has a bright spot from the double-sided epi-polished Al_2_O_3_ single-crystal substrate oriented in c-plane (0001) and the weak arc from silver nanocrystallites with periodicity 3.88 Å and random orientation in space (see Additional file 1). Similar XRD2 patterns were obtained also for 10-nm-thick Ag films deposited at temperatures in the range 200 to 350 K.

**Figure 5:**
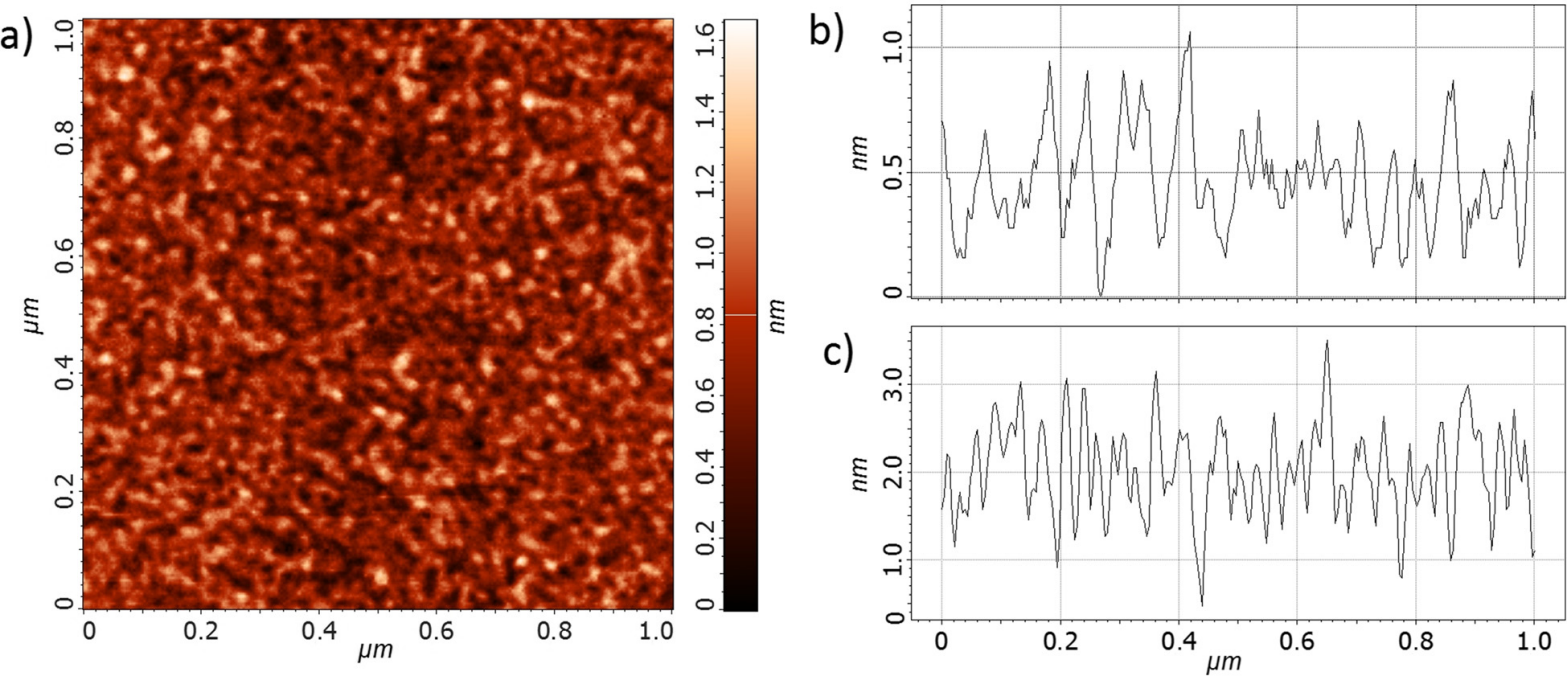
**AFM image and profiles. (a)** AFM image of 10-nm-thick Ag film deposited at 295 K. The lowest ever reported morphology parameters for e-beam deposition technique are as follows: ten-point height value = 1.05 nm, average height = 0.9 nm, and RMS height = 0.22 nm. AFM profiles of **(b)** 10- and **(c)** 30-nm-thick Ag films deposited at 295 K.

Finally, we consider the roughness of very thin silver layers, which are important for construction of hyperbolic metamaterials [26,27] and plasmonic nanolenses [28-32]. Moreover, nanometer-thick Ag films with low surface roughness and fine crystallinity have low electron oscillation damping loss and thus can guide long-range plasmons [33,34]. In the 10-nm-thick Ag film, all three morphology parameters are considerably reduced due to the residual influence of the Ag-Ge surface adhesive force. Figure 5a, b shows a 2D AFM image and a 1D profile of the 10-nm Ag film with the lowest value, achieved with physical vapor deposition, ever reported: RMS = 0.22 nm and ten-point height equal to 1.05 nm. An example of SEM image of the same sample is presented as supporting data in Additional file 2. To illustrate roughness increase with metal film thickness, we show an AFM profile of the 30-nm Ag film in Figure 5c.

**Figure 6:**
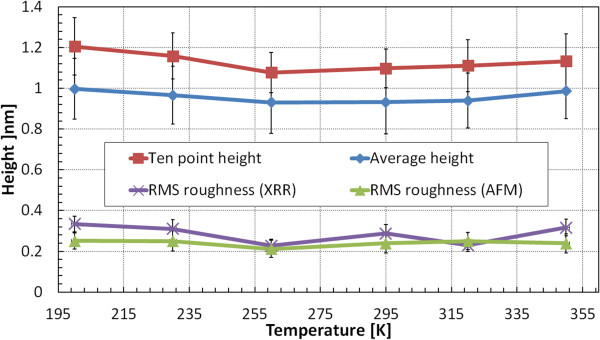
**Ten-point and average height values measured on 3 × 3 μm**^**2 **^**area on 10-nm Ag films.** Thin films were deposited at temperatures in the range 200 to 350 K, and RMS values were measured using both AFM and XRR.

**Figure 7:**
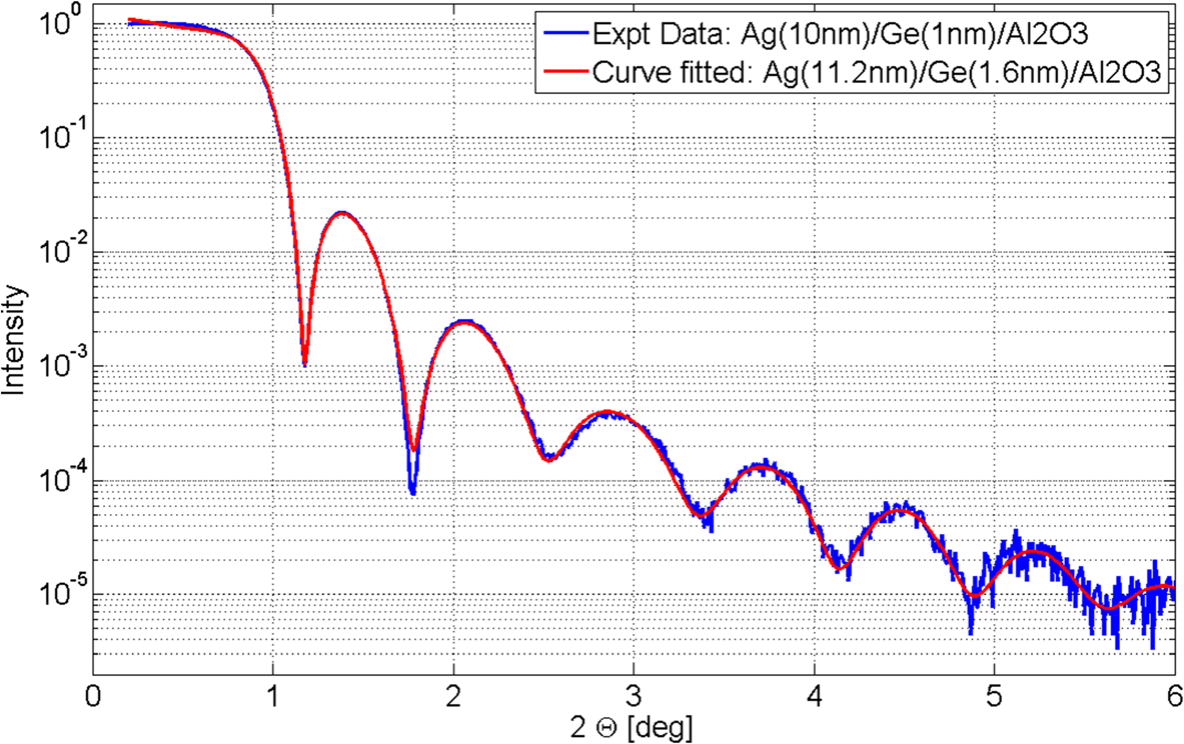
XRR data and fitted model for 10-nm Ag and 1-nm Ge film on sapphire substrate.

In Figure 6, plots of three surface morphology parameters measured on 10-nm Ag films are shown as functions of deposition temperature in the range 200 to 350 K. Similarly to Figure 4, the plots present values averaged from several measurements made on three different samples evaporated at each temperature. Surprisingly, in 10-nm-thick films in the whole range of temperatures 200 to 350 K, adhesive forces between Ag adatoms and Ge wetting layer dominate over cohesive forces in silver. Thus, the temperature-dependent mobility of Ag adatoms does not deteriorate significantly the surface smoothness. RMS roughness values from tapping-mode AFM measurements of 10-nm Ag films are in agreement with those obtained using XRR. An example of XRR data obtained for the 10-nm-thick Ag film deposited on 1-nm Ge interlayer and a fitted model are shown in Figure 7. The average film thickness measured using XRR is 10.9 ± 1.1 nm and differs up to 10% from the values controlled with calibrated quartz weight installed in the vicinity of substrates in the vacuum chamber of the e-beam evaporator. In single-layer structures, e.g., plasmonic silver lenses [28,29], such fabrication inaccuracies should less deteriorate performance than in the case of metal-dielectric-layered flat lenses [30-32].

**Figure 8:**
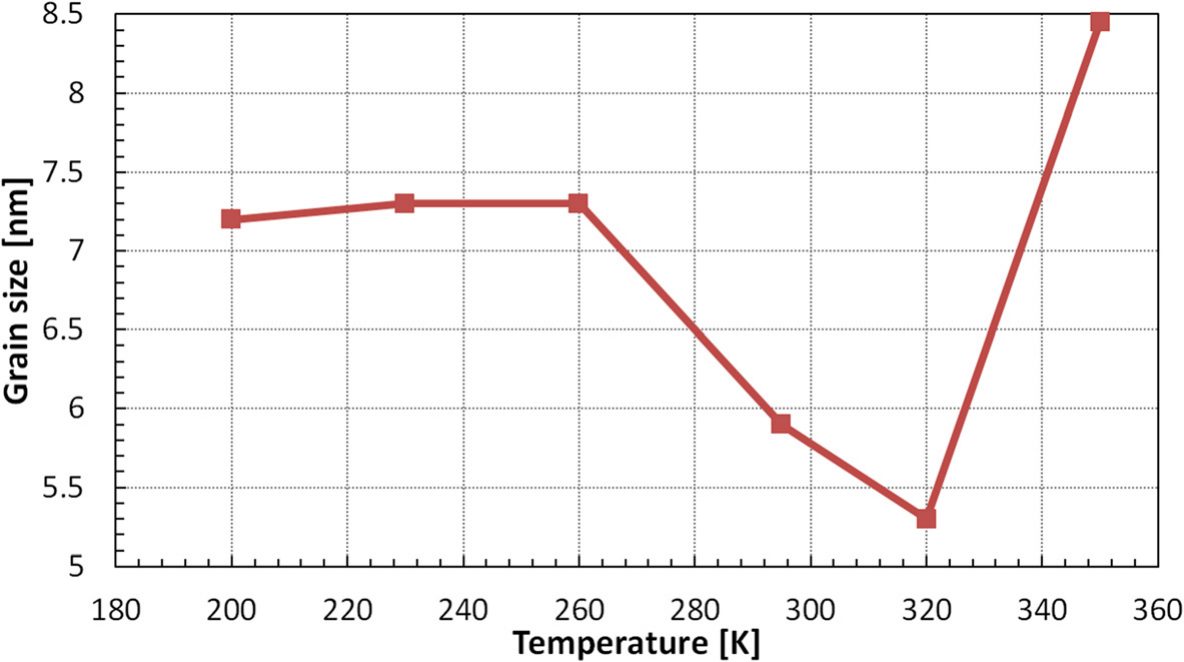
**Grain sizes measured using one-dimensional XRD.** Ag films of 10-nm thickness were deposited at temperatures in the range 200 to 350 K.

At the end, we investigated the interior structure of 10-nm-thick samples using one-dimensional XRD. The dependency between grain size and the substrate temperature is presented in Figure 8. Again, the samples evaporated at temperatures close to RT have the best uniformity.

## Conclusions

A new sublimation-pressure empirical equation valid in the range from 50 K to *T*_t_ = 273.16 K of the triple point helps select the optimum temperature in high-vacuum physical vapor deposition systems. We have demonstrated the possibility to fabricate ultrasmooth metal nanolayers deposited onto epi-polished substrates at the lowest achievable pressure and at such a temperature that the whole dynamic range of both parameters is located on the gas side of the phase-boundary curve of water in a *p*-*T* diagram. The temperature range 230 to 350 K is established as the optimum for deposition of Ag nanolayers using e-beam evaporators. For the 10-nm Ag film on 1-nm Ge interlayer deposited at RT on sapphire substrate, a surface roughness with RMS = 0.22 nm has been achieved. For 30-nm-thick Ag films on sapphire substrate with 1-nm Ge wetting layer, RMS increases up to 0.49 nm. The ten-point height parameter given by extreme local surface features, which reflects scattering properties, has its minimum at 295 K. The achieved reduction of random surface roughness decreases the scattering losses of the propagating SPP wave and thus increases the useful area of metal-dielectric interface in plasmonic devices.

## Abbreviations

AFM: atomic force microscope; PMMA: poly(methyl methacrylate); RMS: root-mean-square; RT: room temperature; SK: Stranski-Krastanov; SPP: surface plasmon-polariton; STM: scanning tunneling microscope; XRR: X-ray reflectometry; XRD2: two-dimensional X-ray diffraction.

## Competing interests

The authors declare that they have no competing interests.

## Authors’ contributions

TS and PW fabricated the samples, made the AFM measurements, and participated in the data analysis. EG made the X-ray measurements. TS wrote the main part of the manuscript. All authors read and approved the final manuscript.

## Supplementary Material

Additional file 1 **Two-dimensional X-ray diffraction (XRD2) pattern of the crystalline 30-nm-thick Ag layer deposited at 295 K.** The central bright spot comes from diffraction on Al_2_O_3_ single-crystal substrate and the weak arc from silver nanocrystallites with periodicity 3.88 Å and random orientation in space. Click here for file

Additional file 2 **SEM image of the 10-nm Ag film on 1-nm Ge interlayer deposited at RT on sapphire substrate.** The 10-nm Ag film has the lowest, ever reported, surface roughness of RMS = 0.22 nm and ten-point height equal to 1.05 nm. Click here for file
