# Supplementary figures and images for: Optimum deposition conditions of ultrasmooth silver nanolayers
Source: Nanoscale Res Lett. 2014 Mar 31;9(1):153. doi: 10.1186/1556-276X-9-153 (PMC4021572; doi:10.1186/1556-276X-9-153)

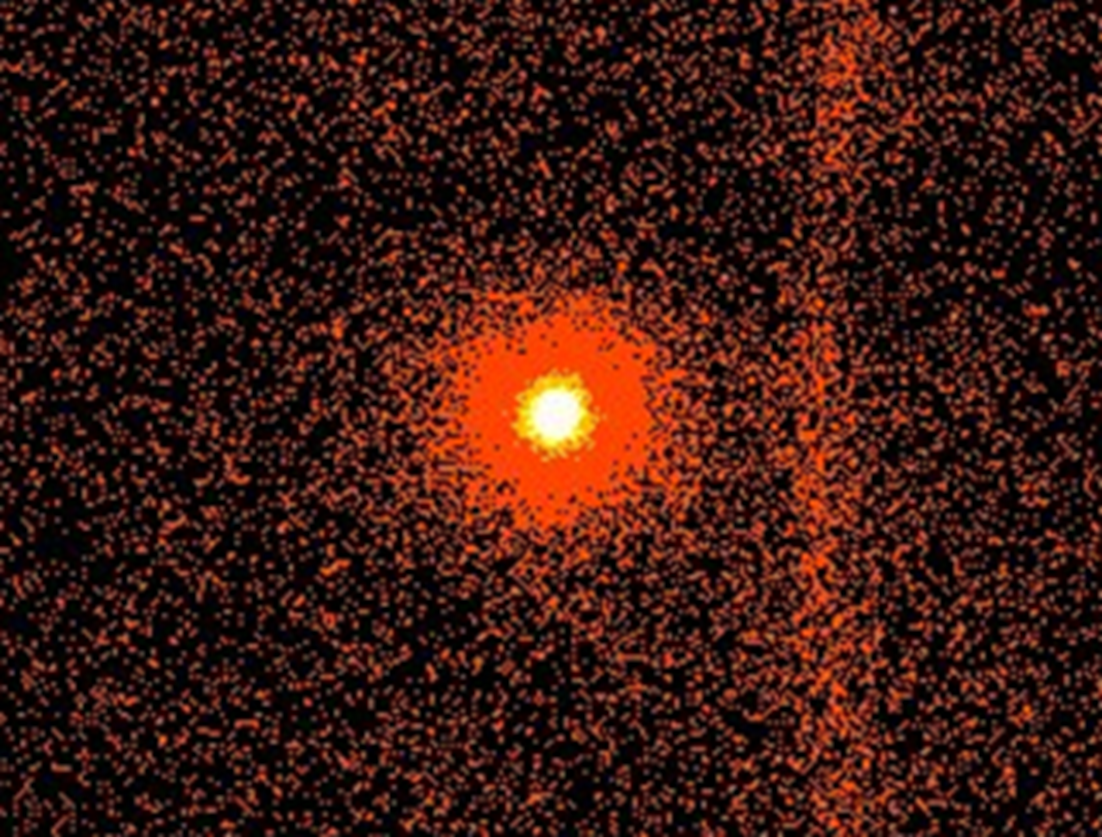

Supplement: Additional file 1 — Two-dimensional X-ray diffraction (XRD2) pattern of the crystalline 30-nm-thick Ag layer deposited at 295 K. The central bright spot comes from diffraction on Al2O3 single-crystal substrate and the weak arc from silver nanocrystallites with periodicity 3.88 Å and random orientation in space. [file 1556-276X-9-153-S1.png]

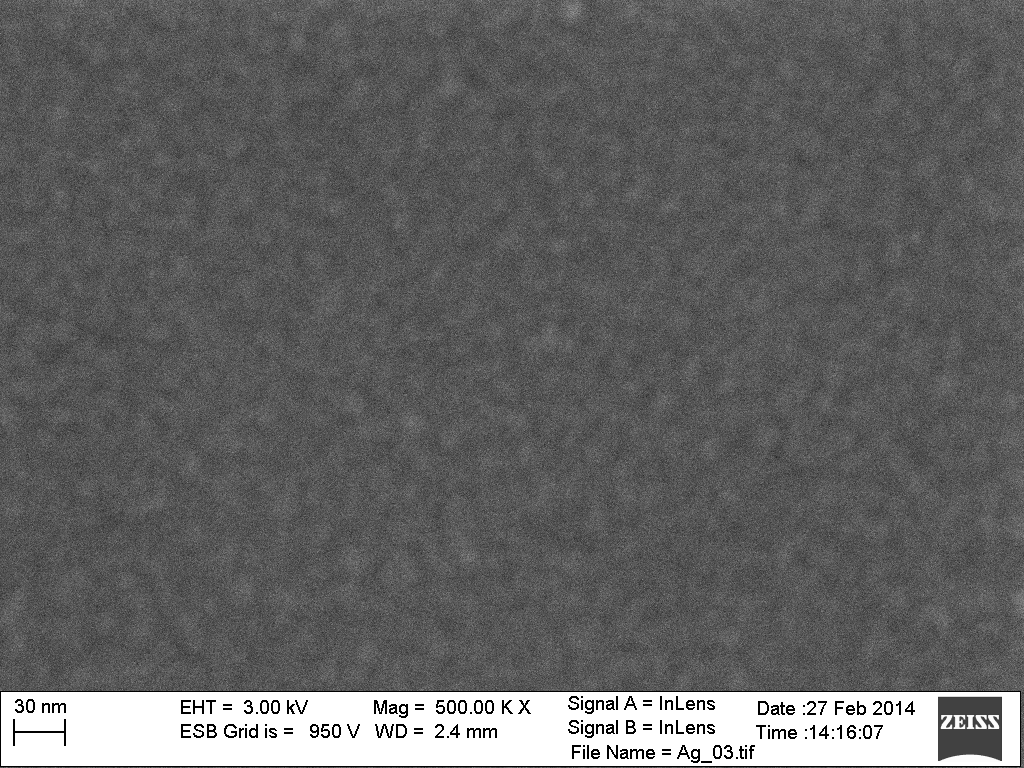

Supplement: Additional file 2 — SEM image of the 10-nm Ag film on 1-nm Ge interlayer deposited at RT on sapphire substrate. The 10-nm Ag film has the lowest, ever reported, surface roughness of RMS = 0.22 nm and ten-point height equal to 1.05 nm. [file 1556-276X-9-153-S2.png]
